# Data-driven psychophysical methods to diversify SIAs and address bias

**DOI:** 10.1007/s12193-026-00472-9

**Published:** 2026-02-05

**Authors:** Valentina Gosetti, Rachael E. Jack

**Affiliations:** School of Psychology and Neuroscience, University of Glasgow, 62 Hillhead Street, Glasgow, G12 8QB Scotland, UK

**Keywords:** Socially interactive agents, Human social perception, Cultural diversity, Bias mitigation, Data-driven modeling

## Abstract

To realize their full potential, Socially Interactive Agents (SIAs) must effectively engage with human users from diverse individual, social, and cultural backgrounds. However, most current SIAs are grounded in White- and Western-centric assumptions, limiting their ability to express and interpret social cues appropriately across cultures. Here, we demonstrate how the data-driven psychophysical method of reverse correlation can help address these limitations by modeling users’ perceptual expectations, preferences, and sociocultural norms and strategically integrating these insights into SIA design. Drawing on examples from our research group, we show how this method could enable SIAs to exhibit social signals that are psychologically grounded, culturally adaptive, and ethnically inclusive. By informing the design of SIA appearance and expressive behavior with empirically derived user models, our approach aims to improve user engagement and trust while contributing to broader efforts to mitigate algorithmic bias, reduce access inequality, and challenge real-world prejudice in both human-AI and human–human interaction contexts.

## Introduction

Socially Interactive Agents (SIA) are fast becoming part of everyday settings, including homes, schools, hospitals, museums, and shopping centres, performing a variety of tasks [1–3]. Many state-of-the-art SIAs have realistic human-like appearance, including individualized identities and dynamic facial expressions, which can enhance user trust, engagement, and experience [4–6]. However, these advances disproportionally benefit a narrow slice of human society—White individuals from Western, Educated, Industrial, Rich, and Democratic (WEIRD) societies [7]. This is because SIAs are often designed using psychological theories and social norms grounded in WEIRD-centric cultural frameworks. For example, facial expressions of happiness commonly used in affective computing [8, 9] and embedded in SIAs [10–12] often feature broad, intense smiles. While interpreted positively in many WEIRD cultures, such expressions can be met with negativity in some Eastern cultures [13, 14]. Similarly, SIA identities often reflect White phenotypes [see [15], for a review] and encode White- and WEIRD-centric ideals of attractiveness and trustworthiness [16]. As a result, SIAs lacking a culturally sensitive social intelligence may violate the expectations of diverse users, reducing their acceptability, effectiveness, and societal value [17].

This WEIRD-centric bias in SIA design reflects longstanding biases in psychological research. Much of what we know about human social perception stems from theories, participant samples, and methods rooted in White and WEIRD populations, which often overlook meaningful cultural, ethnic and individual differences [see [16, 18], for further discussion]. For example, the so-called universal facial expressions of emotion, frequently integrated into SIA design [9–12] are based on WEIRD-specific assumptions that do not generalize reliably across cultures [see [18], for a review]. SIA-based studies confirm that users from different cultural backgrounds interpret emotion signals differently [19]. Similarly, findings on which faces are considered attractive, trustworthy, or dominant are often derived from young, White male faces and WEIRD participants [see [16], for further discussion]. Many of these conclusions also rely on average-based statistical methods, which can obscure individual and group-level variability [20] and inflate the risk of Type I errors [21]. Consequently, current models of human social perception—and the SIA appearances and expressive behaviors that build upon them—fail to capture the breadth of human diversity, limiting their ability to engage meaningfully and equitably with global user populations.

### Consequences and limitations of WEIRD-centric SIAs

White- and WEIRD-centric SIAs pose significant risks to equitable access and benefit. For example, SIAs designed to provide social support for at-risk populations—such as children with chronic illnesses [22] or older adults [23]—must express emotions in culturally appropriate ways to effectively comfort and engage users. When emotional expressions fail to align with users’ cultural expectations, SIA effectiveness could be severely diminished. Similarly, SIAs deployed in public settings, such as museums [1], must be perceived as knowledgeable and trustworthy by diverse audiences. Perceived credibility is influenced by appearance, including ethnic congruence with the user [24] and culturally specific markers of trustworthiness, such as larger eyes and upturned mouth corners [25]. To serve diverse populations effectively, users should have access to customizable SIAs that can display different ethnicities and culture-specific social signals. However, because most SIAs are designed according to White- and WEIRD-centric norms and expectations of appearance and expressive behaviour, they often fail to meet the needs of users outside these strict cultural frameworks.

This limitation also restricts the potential of SIAs to address real-life bias and prejudice in domains such as healthcare [26], the justice system [27], and employment [28]. As ethnically, culturally and gender diverse SIAs evoke similar human biases as real humans do [29, 30], they could be used to challenge implicit biases if incorporated into targeted interventions designed to reduce stereotyping [31] or improve intercultural communication [32]. However, the effectiveness of such interventions is constrained by current SIA models of facial appearance and expressive behavior, which remain grounded in White and WEIRD-centric norms. Addressing this limitation requires broadening the foundational knowledge of human social perception to include diverse cultural, ethnic, and individual perspectives.

To address these challenges, we present a data-driven psychophysical modelling method called reverse correlation, which provides a direct path for equipping SIAs with the social intelligence necessary to improve access equity and mitigate bias and prejudice. Drawing on research from our group, we demonstrate how this approach has been used to develop ethnically and culturally diverse models of social signals, including facial expressions of emotion [33] and facial features associated with social attributes such as trustworthiness and attractiveness [34, 35]. We also provide evidence that these perceptual models can enhance the social signalling capabilities of SIAs [17, 36], including their human-likeness and cultural expressivity, resulting in improved user engagement within and across cultures. Notably, these models have been employed by researchers outside our group to study the effects of gaming on emotion perception [37], neural processing of facial expression features [38], and the influence of face ethnicity on pain perception [39], demonstrating the versatility and broader applicability of our approach. Finally, we propose how future research could extend these tools to personalize SIAs for targeted interventions, such as addressing implicit bias in the workplace, with a view of encouraging new collaborations with SIA designers.

Given the central role of the face in social interactions, we focus here on facial cues, including both dynamic expressions and static features, such as shape and complexion. However, the reverse correlation method is highly generalizable and can be applied to a wide range of stimulus features—including vocal signals [40] and body movements [41]—as well as diverse response measures, such as brain activity [42], animal behavior [43], and outputs from deep neural networks [44].

## Data-driven psychophysical modelling methods to diversify SIAs

**Fig. 1:**
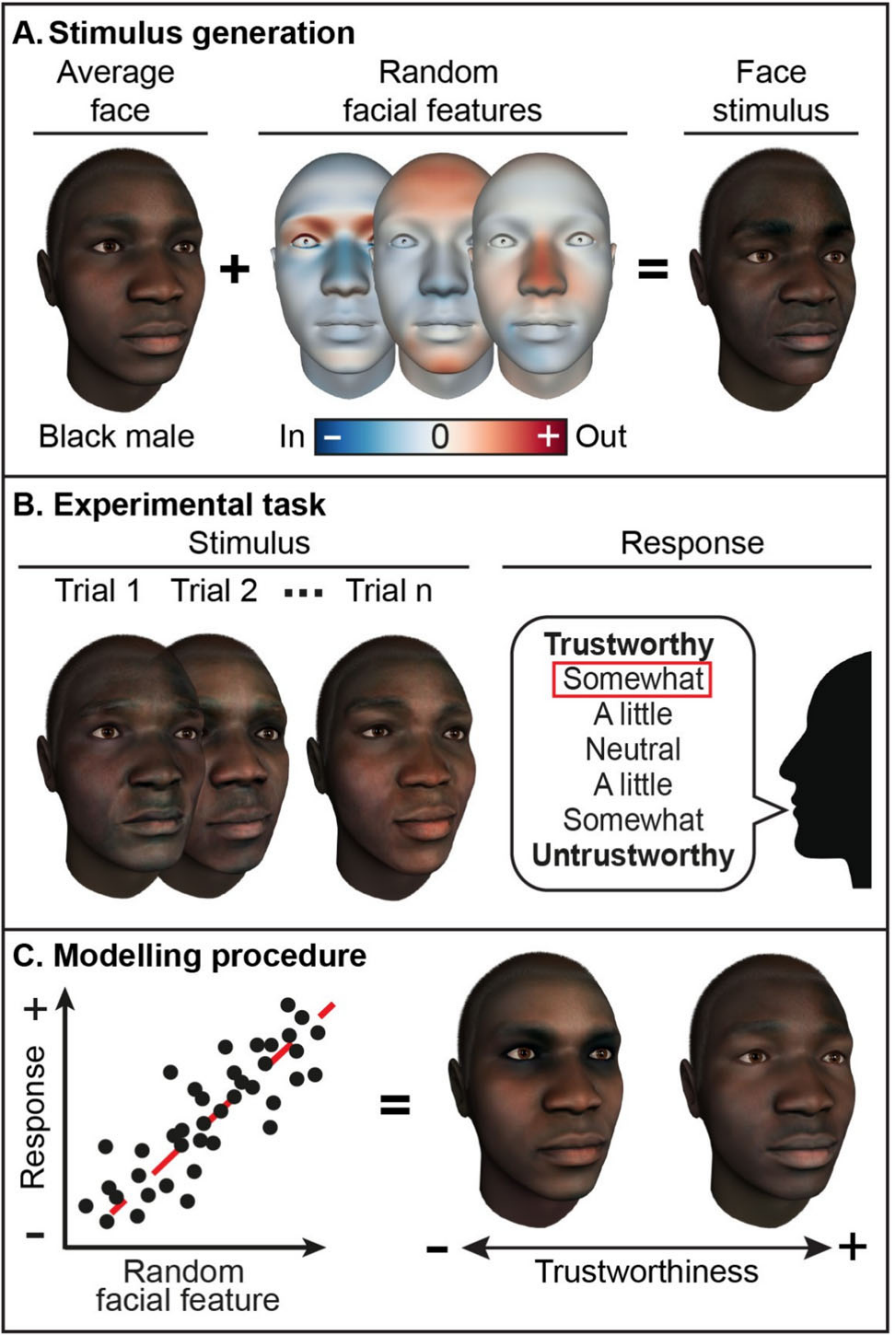
Illustration of procedure to model the facial features driving social perception using reverse correlation. **A** Stimulus generation. **B** Experimental task. **C** Modeling procedure

Reverse correlation is a data-driven modelling method that maps the relationship between objectively measurable stimulus features and subjective perceptions [45, 46]. Unlike traditional theory-driven approaches [47], which often rely on pre-defined assumptions about which features drive responses, reverse correlation explores the stimulus space in an agnostic manner [46, 48]. This serves two key purposes: (i) it enables the discovery of novel perceptual effects without theoretical constraints [49]; and (ii) it isolates the specific stimulus features that drive individual perceptions by statistically linking controlled stimulus variations to user responses [46]. Here, we show how this approach can be used to model the facial features that drive the perception of different social signals. To fix ideas, we focus on one representative example: trustworthiness (Fig. 1).

Stimulus generation is the first step (Fig. 1A). This requires a large set of faces that: (i) are not constrained by prior assumptions, (ii) reflect the natural diversity of human faces, and (iii) allow precise feature control. We achieve this using a high-fidelity 3D generative model of the human face, built from high resolution 3D captures of real people varying in ethnicity, sex, and age [50, 51]. This model has been validated through out-of-sample testing and offers expressive power across both static and dynamic facial features [see [50], for examples]. The model can also generate facial movements—based on Action Units defined in the Facial Action Coding System [52]—using 3D captures of trained human performers [see [50, 51], for more details].

This model therefore enables us to generate face stimuli that satisfy the three criteria outlined above. Specifically, each face stimulus is (i) agnostically sampled—that is, generated without making assumptions about which facial features might represent trustworthiness to whom; (ii) derived from a large, diverse database of real faces that captures the broad natural variance of human facial appearance; and (iii) mathematically decomposable, allowing fine-grained feature control. To illustrate, consider the example in Fig. 1A. We start with an average face of a young Black African male (left), and apply random variations to static facial features (center)—shown here with inward and outward deviations from the same average face, as indicated by the colorbar. This results in a new, randomly generated—but precisely parameterized—face stimulus (right), ready for use in the experiment [see [50, 51], for more details on the stimulus generation procedure].

Each participant is then presented with the same large set of randomly generated face stimuli and asked to rate each one according to trustworthiness. Faces are perceived as trustworthy only if their features align with the participant’s expectations; otherwise they are rated as “neutral” [46]. For example, in Fig. 1B, the participant rated this face stimulus as “somewhat trustworthy” (highlighted in red), indicating that the facial features in this particular trial matched their expectations of trustworthiness.

Finally, after many such trials (typically *N* = 1,200, though this may vary depending on the specific experimental design), we measure the statistical relationship between the facial features presented across the experiment and each participant’s responses (as shown in Fig. 1C, left). This produces a statistically robust 3D model of the facial features that each participant associates with trustworthiness. For example, the faces in Fig. 1C illustrate the 3D models of the facial features considered untrustworthy-looking (left) and trustworthy-looking (right) by one representative participant. To ensure the generalizability of these findings, each 3D model is subsequently validated using an additional sample of participants from the same cultural or demographic group. Therefore, in contrast to traditional theory-driven methods [47], this data-driven approach leverages each participant’s individual expectations to model and validate the facial features associated with trustworthiness.

This method offers several advantages over traditional approaches [see [46], for further discussion]:

1. *Minimizing experimenter bias*: Agnostic feature generation reduces reliance on prior assumptions that could skew the results. This is particularly valuable when studying social signals across diverse cultures and demographic groups, where existing theory and intuition may be less applicable and could introduce bias [49].
2. *Capturing demographic variance*: The generative model [50, 51] objectively represents natural variability across ethnicity, sex, and age, addressing the White-centric bias in current models of social perception [16]. This enables new insights into the social perception of faces within and across diverse populations. For example, some facial features may be universally perceived as trustworthy, while others vary across cultures or ethnic groups [34].
3. *Isolating causal features*: Third, by controlling the facial features in our stimuli, we isolate and identify the specific features that drive social perception in individuals from a given culture or demographic group. This allows us to avoid problems of feature entanglement and thus derive explainable causal mechanism for social perception [53].
4. *Preserving individual differences*: Finally, by adopting a per-participant analysis approach rather than averaging across participants, we preserve individual variations within the sampled population. This enables the discovery of culturally, ethnically, or otherwise individually relevant differences that are key to capturing the full spectrum of human social signals [20]. Our per-participant modeling approach also enables estimations of the prevalence of effects within the sampled population [54], such as which facial features are widely perceived as trustworthy, and which vary across individuals or cultures.

Using this approach, we can build 3D dynamic models of culturally and psychologically valid social signals, reliably eliciting responses from individuals within a specific cultural or demographic group. Our group has applied this method to model a wide range of social signals, including facial expressions of emotions [33], and facial features associated with beauty [35], trustworthiness [34], and social class [55]. In the following section, we present direct examples of how this approach has led to new insights into human social signals. We focus on past work that has revealed both similarities and differences in the perception of facial features across groups. For each example, we discuss the implications for SIA design and highlight opportunities to use these insights to tackle real-life bias and prejudice.

## Recent advances and applications

### Modeling cultural diversity in facial expressions

Facial expressions are powerful social signals regularly used to communicate emotions and influence the behavior of others. For example, people tend to approach somebody who looks happy, avoid somebody who looks angry, and comfort somebody who looks sad. Influential psychological theories have long proposed that six basic emotions—happiness, surprise, fear, disgust, anger, and sadness—are each expressed through distinct, universally recognized facial expressions [47]. However, mounting evidence now challenges the universality of these expressions [56, 57]. Recognition of fear, surprise, anger, and disgust is often significantly poorer (i.e., lower accuracy) in East Asian cultures compared to Western cultures [58]. Moreover, standardized facial expressions are evaluated differently across cultures—for example, broad smiles, which are commonly associated with intelligence, attractiveness, and competence in Western societies, may instead be associated with naivety, mistrust, or incompetence in Eastern cultures [14, 59].

**Fig. 2:**
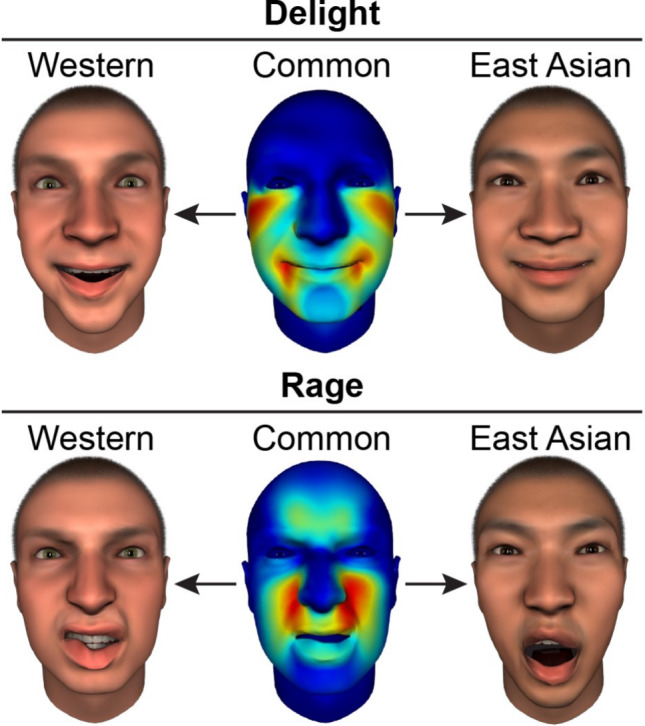
Color-coded faces in the center show the culturally common latent expressive patterns associated with the complex emotions delight (top) and rage (bottom). On either side, Western (left) and East Asian models (right) of these facial expressions are shown

To develop a more culturally valid model of facial expressions of emotion, Jack et al*.* [33] used the reverse correlation approach described above to model the dynamic facial expressions of over 60 emotions in participants from Western European and East Asian cultures. Analyses revealed that—contrary to the long-standing assumption of six basic universal emotional expressions—a culturally shared set of four latent expressive patterns underlies emotion expression across both cultures. These latent patterns primarily represent valence (positive vs negative) and arousal (high vs low). Figure 2 shows two such expressive patterns, which include: smiling and cheek raising (top row), associated with high-valence and high-arousal emotions such as “delight”; and nose wrinkling and teeth baring (bottom row), associated with low-valence and high-arousal emotions such as “rage”.

While these expressive patterns were shared across cultures, further analyses revealed culturally specific “accents”—modulations of facial expressions that align with prior findings on cultural variability in emotion signaling [56, 60]. Therefore, the 60 + modeled facial expressions of emotion can be expressed as weighted linear combination of the four culturally shared latent expressive patterns, plus culture-specific accents. For example, as shown in Fig. 2, facial expressions of”delight” include smiling and cheek raising in both cultures plus raised eyebrows and mouth gaping in Western culture, whereas in East Asian culture the facial expression remains more serene. Similarly, facial expressions of”rage” include shared elements of nose wrinkling and teeth baring plus scowling in Western culture and mouth gaping in East Asian culture.

Using the same approach, related work has identified culturally specific similarities and differences in facial expressions used for back channeling responses [e.g., interested, confused [36]], as well as experiences of pain and pleasure [61].

These examples demonstrate the utility of the above-described reverse correlation approach in deriving culturally valid facial expressions, with direct implications for psychological theory and the design of culturally sensitive SIAs. To validate the applied value of these models, our group replaced a SIA’s standard “universal” facial expressions of basic emotions with culturally valid models derived from Western European and East Asian participants [17, 61]. This modification led to a significant increase in emotion recognition accuracy among East Asian users compared to the traditional “universal” facial expressions [17]. Moreover, both Western and East Asian participants perceived these culturally valid models as more human-like compared to traditional models [17, 62].

These promising results suggest that culturally grounded models can improve the realism and accessibility of SIAs across diverse populations. For example, SIAs acting as virtual patients during medical training must display believable facial expressions of pain during physical exams, and appropriately convey sadness and anger when receiving bad news [62]. However, if these facial expressions are not culturally recognizable, the training tool may be ineffective—or worse, may reinforce inaccurate cultural assumptions. Cross-cultural misinterpretations of facial expressions could also reduce adherence to treatment recommendations [63], thereby increasing health risks at both individual and community levels. Finally, SIA-based training systems that reflect WEIRD-centric facial expressions of pain could worsen medical racism [26], where poorer recognition of pain in minority patients could contribute to under-treatment [64]. Therefore, incorporating cultural diversity into SIA design is essential for developing socially competent systems that can equitably engage users across cultural boundaries and contribute to fairer access to emerging technologies worldwide.

Additionally, SIAs embedded with culturally sensitive facial expressions could be used as training tools to enhance cross-cultural communication and facilitate cultural integration. In increasingly multi-cultural societies, interactions between individuals from different cultural backgrounds are becoming more frequent. However, such interactions often risk miscommunication if facial expressions are not accurately interpreted across cultures [18]. Culturally sensitive SIAs could address this challenge by serving as interactive training tools that teach individuals to recognize facial expressions from other cultures and raise awareness of different communication norms. This application presents several benefits over traditional human-led training, where the task could become tedious for trainers and potentially uncomfortable or anxiety-inducing for trainees. Supporting this, SIAs have already demonstrated efficacy in related training contexts, such as improving public speaking skills [65] and preparing users for job interviews [66]. Further, as SIAs can evoke expressive behaviors such as facial mimicry in human users [67], these systems could also be used to train individuals to produce culturally appropriate facial expressions—an important step towards smoother integration into new cultural environments. Thus, equipping SIAs with culturally adaptive facial expressions offers promising opportunities to improve cross-cultural communication, promote cultural awareness, and support the broader goals of cultural integration and social cohesion.

Together, in contrast to traditional theory-driven methods [47], the data-driven approach described here and used by Chen et al*.* [17], Jack et al*.* [33], and Chen et al*.* [61] enabled the discovery of both shared and culture-specific facial expressions. These findings advance psychological theory by offering a more nuanced understanding of how emotional expressions vary across cultures. At the same time, they provide a critical foundation for the development of culturally adaptive SIAs, with direct applications across diverse personal and professional contexts where effective social communication is essential.

### Modeling cultural diversity in beauty preferences

Physical attractiveness exerts a powerful influence on social interactions—for example, attractive individuals are often judged as more likable and competent [68, 69], leading to significant personal and professional advantages [28, 70]. Longstanding theories have posited that beauty is universal, emphasizing preferences for faces that are closer to the population average [71] and have exaggerated sexually dimorphic features [72]. However, emerging evidence challenges these assumptions by highlighting substantial cultural and individual variations in beauty preferences [73, 74].

**Fig. 3:**
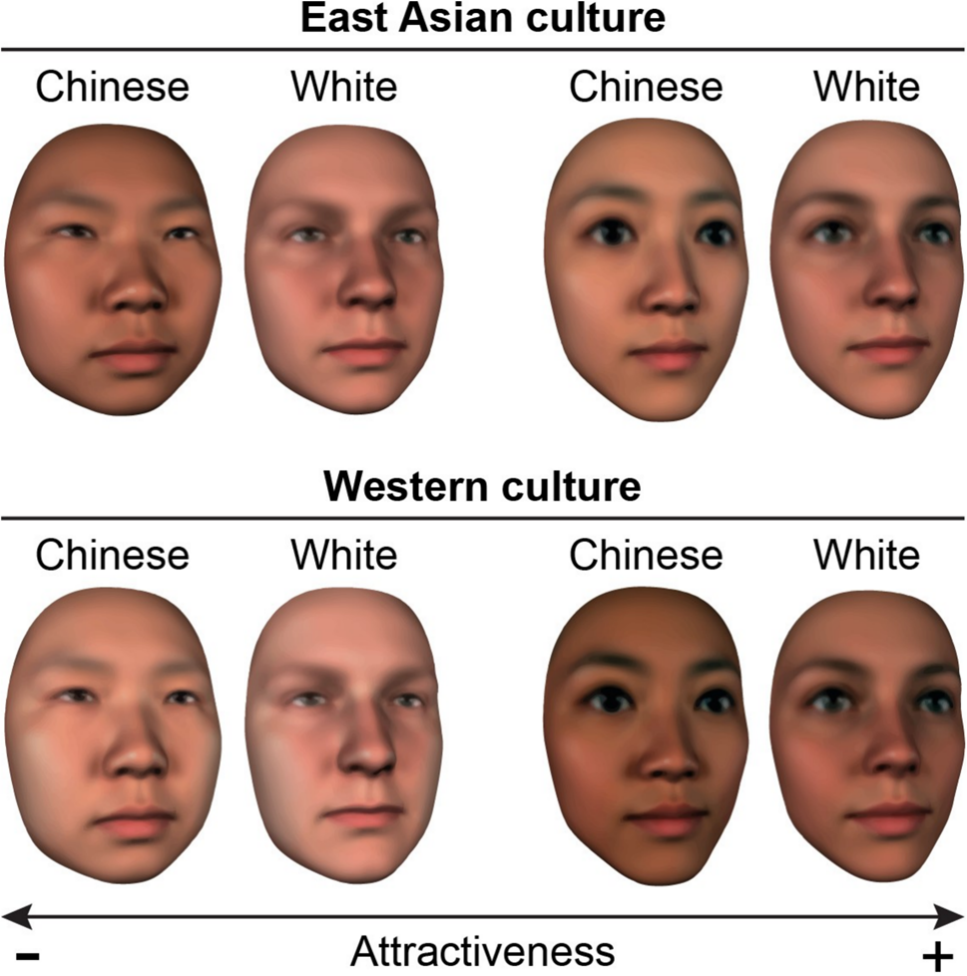
Examples of Chinese and White female faces judged as unattractive (left) and attractive (right) in East Asian (top) and Western culture (bottom)

To systematically examine these variations, Zhan et al*.* [35] used the above-described reverse correlation approach to model the facial features associated with attractiveness in East Asian and Western European male participants judging both same and other-ethnicity female faces. Across all conditions, results revealed that attractive faces differed from theoretical norms: they were neither average-looking nor marked by pronounced sexual dimorphism. Instead, both cultural groups favored faces that are smaller with larger, rounder eyes than the population average. Importantly, preferences also aligned with culturally dominant beauty ideals: East Asian participants favored lighter skin tones, while Western participants preferred darker complexions regardless of the ethnicity of the face (see Fig. 3 for examples).

Therefore, using this agnostic, data-driven approach, Zhan et al*.* [35] demonstrated that longstanding psychological theories fail to fully capture cross-cultural perceptions of beauty. By directly comparing beauty models across cultures, Zhan et al*.* [35] revealed that while certain facial features—such as smaller faces with larger eyes—are considered beautiful across cultures, cultural norms significantly influence beauty perceptions beyond simple recognition of ethnic group membership. These findings not only advance psychological theory but also have practical implications for SIA design.

For example, consider SIAs used in educational settings to act as learning aids and role models [3, 75], particularly for students from underrepresented groups [e.g., women in STEM; [77]]. To motivate students and improve learning outcomes, SIAs must be likable and persuasive while also resonating with the student’s identity [5]. Evidence shows that SIA attractiveness influences both user preference [76, 77] and agent persuasiveness [78]. Furthermore, SIAs that appear to belong to the same demographic group as the user are more effective at influencing behavior [i.e., ingroups; [81]]. By incorporating culturally sensitive beauty standards into the design of demographically diverse SIAs, designers could optimize both in-group recognition and perceived attractiveness to enhance student motivation and engagement. In doing so, the educational benefits of classroom SIAs could be more equitably experienced by users from diverse cultural and demographic backgrounds.

An additional advantage of incorporating culturally sensitive beauty standards into SIA design is their potential to address gender biases in professional settings. Specifically, evidence shows that perceptions of attractiveness often interact with perceptions of competence to influence hiring decisions—particularly disadvantaging female applicants, but not their male counterparts [28]. To mitigate this bias, hiring managers could be trained using female SIAs that vary in culturally sensitive beauty standards but exhibit the same level of competence, as demonstrated through consistent displays of knowledge and skills. This application is particularly promising given emerging psychological evidence suggesting that direct behavioral evidence—such as demonstrated expertise—can override initial appearance-based judgments [80]. Moreover, by leveraging culturally valid beauty models, this training could be effectively implemented in both local and multinational organizations to combat hiring biases across cultural contexts. Thus, designing culturally sensitive SIAs offers a powerful tool not only for improving user experience but also for challenging biases in real-world decision-making processes.

Together, and in contrast to previous White- and WEIRD-centric investigations of beauty perception [71, 72], the data-driven method used by Zhan et al*.* [35] enabled the objective modeling of culturally specific beauty preferences. By revealing clear cross-cultural differences in perceptions of attractiveness, this method advances psychological theory and informs the design of SIAs toward a more inclusive and representative understanding of human social perception.

### Modeling ethnic variance in perceptions of trustworthiness

Humans regularly make spontaneous judgments about who to trust or avoid based on facial appearance [81]. Prominent psychological models posit that these judgments are driven by specific facial features—for example, smaller, rounder faces with larger eyes and full, upturned lips tend to be perceived as trustworthy, whereas longer, narrower faces with a pronounced brow ridge and tight lips are associated with dominance [82]. While emerging evidence suggests that the ethnicity of a face influences these social judgments [83], current models are based almost exclusively on White faces [16]. As a result, it remains unknown whether face ethnicity influences the specific facial features that people use to make these social trait judgments. To address this gap, Gosetti et al*.* [34] used the above-described data-driven approach to model the facial features that drive perceptions of trustworthiness and dominance across ethnically diverse faces—specifically, Black African, East Asian, and White European—in White Western participants. Importantly, all tested faces (*N* = 1,200 per face ethnicity) comprised the same facial feature variations across ethnicities to enable direct comparisons.

**Fig. 4:**
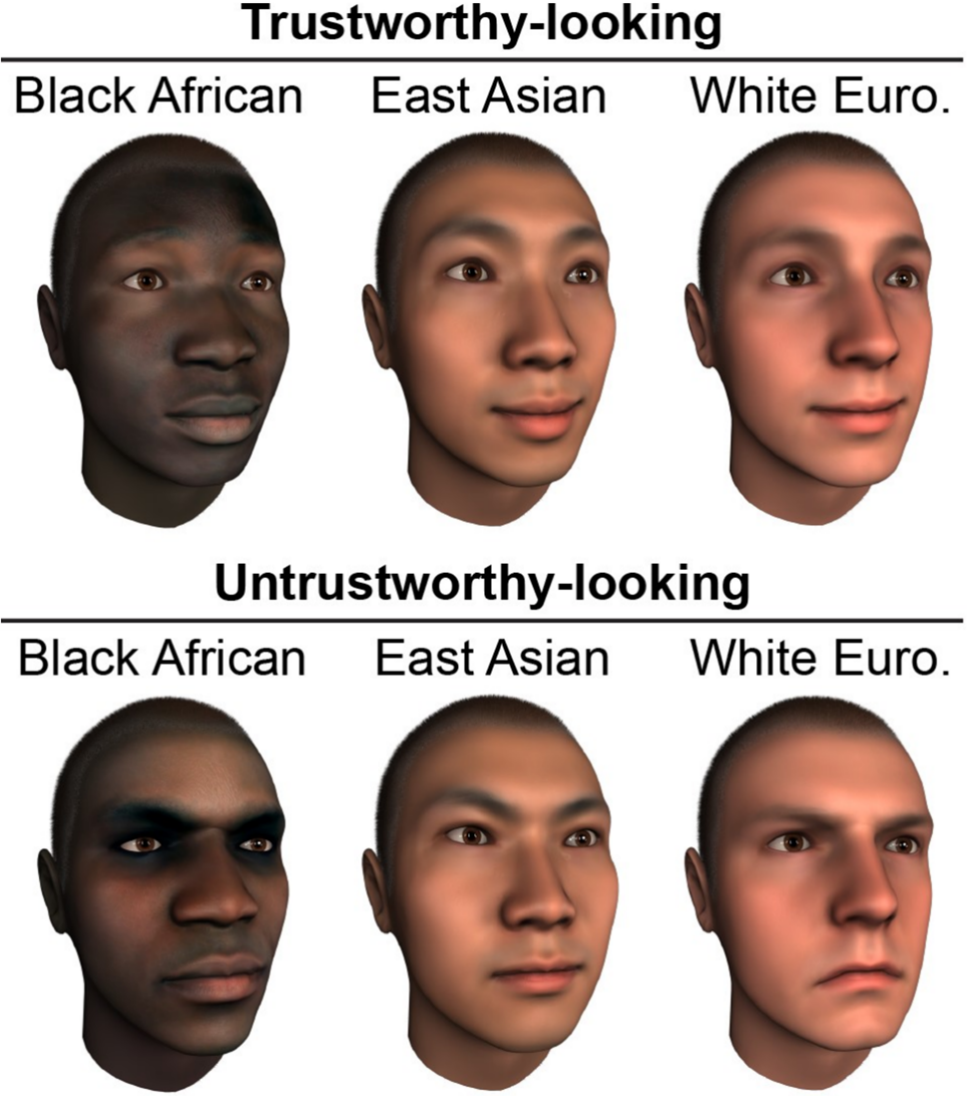
Examples of trustworthy-looking (top) and untrustworthy-looking (bottom) Black African, East Asian, and White European male faces

Results showed that while prominent models [82] accurately capture the facial features associated with trustworthiness and dominance in White faces, they fail to generalize to Black African or East Asian faces. Rather, in line with research on ethnic stereotyping [84, 85], participants perceived faces of other ethnicities as more trustworthy when their salient ethnic phenotypic features were attenuated. Specifically, Black African faces judged as trustworthy tended to have a smaller mouth and lighter eye regions than the average Black African face, whereas East Asian faces judged as trustworthy were characterized by a narrower face with flatter cheekbones than the average East Asian face (see Fig. 4 for examples). In contrast, dominant-looking faces across all three ethnicities shared broadly similar facial features, though the strength of these features varied by ethnicity. Finally, preliminary findings from a parallel study involving East Asian participants suggest that both the cultural background of the observer and the ethnicity of the target face shape judgments of trustworthiness and dominance. This highlights the dual influence of participant culture and face ethnicity on social face perception.

Thus, by agnostically testing the influence of ethnically diverse faces on perception, Gosetti et al*.* [34] demonstrated that fundamental social judgments that are key to deciding who to trust or avoid are systematically biased by face ethnicity. Specifically, the above-described data-driven approach enabled Gosetti et al*.* [34] to objectively reveal *how* face ethnicity influences social perception—a finding previously obscured by the limited ethnic variability of previous work [16]. These findings extend prominent psychological models [82] by incorporating ethnic variability into the mechanisms of social face perception.

Importantly, this work also offers concrete applications for the design of ethnically sensitive SIAs. Prior studies show that user perceptions of likability, human-likeness, and competence are influenced by the SIA’s perceived ethnicity [79, 86, 87]. To mitigate these biases, SIA design could integrate facial features associated with trustworthiness in ways that are culturally flexible and ethnically sensitive. This could enhance the effectiveness and representativeness of SIAs deployed in diverse roles such as academic tutors, museum guides, and marketing agents. Such designs may also foster a sense of identification among user from underrepresented groups, thereby enhancing the potential of SIAs to act as aspirational role models [77]. In this way, embedding ethnic and cultural diversity into SIA design can support more equitable user engagement and reduce disparities in the societal benefits of these technologies.

Finally, because the data-driven approach outlined here can identify the specific facial features that underpin biased judgments—as demonstrated by Gosetti et al*.* [34]—it holds promise for informing SIA-based interventions aimed at tackling prejudice and facilitating cross-ethnicity interactions. To illustrate, modern workplace environments are increasingly diverse. While some evidence suggests that interracial contact alone can help mitigate existing biases [88], a considerable body of work underscores the persistence of implicit racial bias in professional settings [89, 90]. These findings highlight the need for more targeted and effective interventions [91]. To this end, SIAs offer a promising platform for bias reduction. For example, demographically diverse SIAs could be programmed to not only appear trustworthy and competent—based on the culturally and ethnically sensitive models described here—but also demonstrate these traits through meaningful actions, such as participating constructively to brainstorming sessions [92]. Additionally, interventions could include SIAs designed to reflect users’ own biased expectations of ethnically diverse individuals, offering a safe and controlled environment in which users can confront and reassess their implicit stereotypes. This strategy aligns with recent successes in reducing facial stereotyping of White faces through similar training efforts [31].

Together, these findings underscore the critical importance of incorporating cultural and ethnic diversity into models of human social perception—including those that capture the nature of social biases. By moving beyond the limitations of WEIRD-centric and ethnically homogeneous research, this approach enables a more accurate and inclusive understanding of how humans perceive and interact with one another. In turn, such advancements contribute to the refinement of psychological theory and offer powerful opportunities to enhance the design of SIAs. Specifically, by embedding these models into SIA design, we can develop tools that are more socially competent, representative, and effective at challenging prejudice in real-life settings.

## Ethical considerations in diversifying SIAs

Representing human diversity in SIAs has the potential to both improve access equity and mitigate real-life bias and prejudice. For example, adapting SIA design to reflect the ethnic and cultural background of the user could enhance both user engagement [24] and SIA effectiveness [79]. However, such personalized designs should comply with data protection laws and thus be controlled by the user as an optional setting. For example, SIAs employed as museum guides [1] could represent a range of ethnic and cultural identities that visitors could select as desired. Such user-driven customization would not only circumvent potential data risks but also promote representation and empowerment for underrepresented user groups [77].

Additionally, as with SIAs more broadly, culturally and ethnically sensitive SIAs could be exploited to manipulate user behavior in unethical or harmful ways. For example, adapting only the physical appearance of SIAs to match users’ cultural or ethnic expectations while retaining underlying behaviors shaped by WEIRD design assumptions could create a misleading impression of cultural sensitivity. This may foster misplaced trust and inadvertently ‘cheat’ users into believing that the agent understands them more deeply than it does. Moreover, culturally attuned SIAs could function as ‘super-stimuli,’ leveraging culturally idealized traits of attractiveness, familiarity, or trustworthiness to gain a covert advantage in human persuasion. Such affordances raise concerns about manipulation, deception, autonomy, and fairness, particularly in commercial or political contexts.

As such exploitative techniques already exist [95] and are largely outside the control of researchers, raising awareness of these applications and their ethical implications remains a central responsibility. Echoing ongoing discussion on the ethics of Human-Robot Interactions and Artificial Intelligence [96, 97], we stress the need for continued cross-disciplinary collaboration among psychologists, computer scientists, ethicists, sociologists, and the general public to ensure that safeguards against such exploitative uses are considered wherever possible.

Overall, while diversifying SIAs requires careful ethical consideration, we maintain that the potential benefit of designing SIAs with human diversity in mind outweighs these risks.

## Conclusion

Here, we have demonstrated how a perception-based, data-driven approach can objectively capture the perceptual expectations, cultural norms, and social biases of diverse user groups. Drawing on concrete examples from our research group, we have illustrated how this method can inform the design of SIAs capable of engaging users across a wide spectrum of cultural and demographic backgrounds. These insights have direct implications for improving the accessibility, effectiveness, and social equity of SIAs, particularly in combating bias and prejudice. Ultimately, we aim to inspire greater collaboration between psychologists and computer scientists in the shared pursuit of understanding, representing, and respecting the broad natural diversity of human behavior.

## Data Availability

Not applicable.
